# Monitoring circulating tumor DNA by analyzing personalized cancer-specific rearrangements to detect recurrence in gastric cancer

**DOI:** 10.1038/s12276-019-0292-5

**Published:** 2019-08-08

**Authors:** Young-Woo Kim, Young-Ho Kim, Yura Song, Han-Seong Kim, Hye Won Sim, Shiv Poojan, Bang Wool Eom, Myeong-Cherl Kook, Jungnam Joo, Kyeong-Man Hong

**Affiliations:** 1 Center for Gastric Cancer, National Cancer Center Hospital, 323 Ilsan-ro, Ilsandong-gu, Goyang-si, Gyeonggi-do Republic of Korea; 2 Cancer Biomedical Science, National Cancer Center Graduate School of Cancer Sience and Policy, 323 Ilsan-ro, Ilsandong-gu, Goyang-si, Gyeonggi-do Republic of Korea; 3 Research Institute, National Cancer Center, 323 Ilsan-ro, Ilsandong-gu, Goyang-si, Gyeonggi-do Republic of Korea; 4 Department of Pathology, Inje University Ilsan Paik Hospital, Ilsanseo-gu, Goyang-si, Gyeonggi-do Republic of Korea

**Keywords:** Cancer genomics, Next-generation sequencing, Cancer screening

## Abstract

Circulating tumor DNA (ctDNA) has emerged as a candidate biomarker for cancer screening. However, studies on the usefulness of ctDNA for postoperative recurrence monitoring are limited. The present study monitored ctDNA in postoperative blood by employing cancer-specific rearrangements. Personalized cancer-specific rearrangements in 25 gastric cancers were analyzed by whole-genome sequencing (WGS) and were employed for ctDNA monitoring with blood up to 12 months after surgery. Personalized cancer-specific rearrangements were identified in 19 samples. The median lead time, which is the median duration between a positive ctDNA detection and recurrence, was 4.05 months. The presence of postoperative ctDNA prior to clinical recurrence was significantly correlated with cancer recurrence within 12 months of surgery (*P* = 0.029); in contrast, no correlation was found between cancer recurrence and the presence of preoperative ctDNA, suggesting the clinical usefulness of postoperative ctDNA monitoring for cancer recurrence in gastric cancer patients. However, the clinical application of ctDNA can be limited by the presence of ctDNA non-shedders (42.1%, 8/19) and by inconsistent postoperative ctDNA positivity.

## Cancer: Tell-tale signs of recurrence

Fragments of tumor DNA, or circulating tumor DNA (ctDNA), in blood can help predict stomach cancer recurrence within 12 months of surgery. Kyeong-Man Hong at the National Cancer Center, in Goyang-si, South Korea, and colleagues, carried out whole genome sequencing of stomach tumor samples from 25 patients to identify personalized cancer-specific rearranged DNA sequences. When they used this information to monitor ctDNA in blood samples obtained after surgical removal of the tumor, they found a significant correlation between the presence of ctDNA and cancer recurrence. In most cases, ctDNA was detected around four months prior to clinical recurrence, highlighting the potential usefulness of ctDNA monitoring. The lack of correlation between ctDNA levels and tumor size suggests that further research into the factors determining ctDNA levels is needed.

## Introduction

Currently, the standard methodologies for monitoring cancer recurrence in patients with gastric cancer after surgical resection are radiographic imaging and peritoneal cytologic examination^1,2^, but these methods lack the sensitivity required for detecting micrometastatic tumors. Circulating tumor DNA (ctDNA) is the fragment of genetic material shed from necrotic or apoptotic tumor cells, thereby introduced into systemic circulation, and found in the cell-free component of blood^3–5^. Recently, ctDNA has emerged as a candidate biomarker for screening cancer patients^6^, for monitoring cancer recurrence^7,8^, and for determining somatic mutations in cancer patients^9,10^.

The clinical significance of ctDNA monitoring for cancer recurrence has been suggested mostly by the use of next-generation sequencing (NGS) for mutation detection^7,8^. However, ctDNA monitoring for cancer recurrence after radical resection of the primary tumor might be difficult as the tumor will be <1 cm in diameter and not clinically detectable via imaging, which might mean that the amount of ctDNA shed is much lower than the detectable limit. Currently, ctDNA mutations are employed in most studies^2,4,7,9,11–13^; however, the present study employed cancer-specific rearrangements to enhance the sensitivity and/or specificity of ctDNA monitoring for cancer recurrence.

The main aims of the present retrospective study were as follows: (1) to conduct a feasibility test for the detection of low-level postoperative ctDNA in serially collected blood samples in the early phases of clinical recurrence in gastric cancer patients who had undergone surgical resection of the primary tumor, and (2) to evaluate the usefulness of postoperative ctDNA for monitoring cancer recurrence. For these purposes, we retrospectively and preferentially enrolled patients with recurrence and available serial plasma samples obtained up to 12 months after curative surgical resection as well as available frozen primary tumor samples. Personalized cancer-specific rearrangements after analyzing the rearranged sequences from whole-genome sequencing (WGS) in gastric cancers were employed to monitor ctDNA in serially collected plasma samples.

## Materials and methods

### Patient selection

Plasma samples were prepared from whole blood the day before surgery and postoperatively at 1, 3, 6, 9, and 12 months after surgical resection of the primary cancer. Fresh-frozen paired tumor and normal tissues were obtained from the Tissue Bank of the National Cancer Center, Korea. All of the patients had been diagnosed with stage II, III, or IV gastric cancer according to the seventh edition of the American joint committee on cancer TNM staging system, and their clinical information is summarized in Table 1 and S1. The use of plasma and tissue samples for the present study was approved by the Institutional Review Board of the National Cancer Center, Korea (NCC2014-0025), and all methods were performed in accordance with the relevant guidelines and regulations. Informed consent from all participants in the present study was obtained during our previous study (NCCTS-04-105) to use their plasma, from the Tissue Bank to use frozen tissues, and waived for the present study.

**Table 1:** Analysis of personalized cancer-specific rearranged sequences in gastric cancer patients

ID	Sex	Age	Recurrence status	Stage (TNM)	Rearranged sites in WG-NGS	Primers designed for PCR sites	Cancer-specific PCR	Confirmed by Sanger sequencing	Final validated sites	PreOp ctDNA	PostOp ctDNA	Lead time (months)
GC1	M	68	R	IIIA (T3N2M0),	48	12	7	4	4	–	–	
GC4	M	67	R	IIIC (T4aN3bM0)	44	7	3	3	3	+	+	9.4
GC6	M	64	R	IIIB (T3N3aM0)	3	3	3	3	3	−	−	
GC7	M	57	R	IIIB (T4aN2M0)	3	3	3	1	1	−		
GC8	M	53	R	IIIC (T4bN3aM0)	8	6	4	3	2	+	+	−1.4
GC9	M	44	R	GIST (T4N0M0)	8	8	3	3	3	+	+	5.0
GC10	M	73	R	IIIB (T4aN2M0)	31	5	3	3	3	−	−	
GC11	M	70	R	IIIB (T3N3aM0)	90	12	6	5	5	+	+	5.6
GC12	M	71	R	IIIC (T4aN3aM0)	3	3	3	2	2	−	−	
GC14	M	53	R	IIIB (T3N3bM0)	25	11	4	2	2	−	+	0.7
GC15	M	42	R	IIIC (T4bN3aM0)	7	7	5	3	2	+	+	3.1
GC17	M	71	R	IV (T4aN3bP1)	16	12	8	5	5	+	+	^a^
GC18	M	41	R	IIB (T3N1M0),	6	5	4	2	2	−	−	
GC21	M	77	R	IIIC (T4aN3aM0)	40	13	6	6	5	−	−	
GC22	M	49	R	IV (T4aN3bP1)	23	9	3	3	3	+	+	^a^
GC31	M	54	N	IIA (T3N0M0),	6	5	4	3	3	+	−	
GC32	M	70	R	IIIC (T4bN2M0)	11	8	8	8	8	+	−	
GC33	M	44	N	IIIC (T4bN3bM0)	6	6	6	6	6	+	−	
GC34	M	63	N	IIIC (T4aN3aM0)	3	3	1	1	1	+	−	
CG35^b^	M	60	N	IIA (T3N0M0)	0	0	0	0	0			
GC2^b^	F	62	R	IIB (T3N1M0)	0	0	0	0	0			
GC3^b^	M	64	R	IIIB (T4aN2M0)	0	0	0	0	0			
GC5^b^	F	72	R	IIIC (T4aN3bM0)	0	0	0	0	0			
GC13^b^	F	73	R	IIIA (T3N2M0)	0	0	0	0	0			
GC23^b^	M	60	R	IIIA (T3N2M0)	3	3	0	0	0			
Total					384	141	84	66	63			

*R* recurrence, *N* nonrecurrence, *PreOp* preoperative, *PostOp* postoperative

− negative ctDNA,+ positive ctDNA

^a^Cases of positive peritoneal seeding

^b^No cancer-specific rearrangement was found in WGS, and no ctDNA monitoring was performed

### Laser-capture microdissection (LCM) from fresh-frozen tumor samples

A pathologist confirmed the gastric cancer cells in each sample and demarcated the tumor areas on hematoxylin and eosin (H&E)-stained slides. To obtain samples consisting of 70% or more tumor cells, the tumor areas were dissected using a laser-capture microdissection (LCM) instrument (Ion LMD, Jungwoo F&B, Korea). The dissected tumor fragments were incubated in 1 M sodium thiocyanate overnight. Subsequently, DNA was isolated using the QIAamp DNA FFPE Tissue Kit (Qiagen, Hilden, Germany). Fresh-frozen tissues were also used as paired normal gastric tissue and underwent DNA preparation after confirmation on H&E-stained slides by a pathologist.

### Library preparation and WGS

Preparation of the sequencing libraries using the TruSeq Nano DNA Sample Preparation Kit (Illumina, San Diego, CA, USA) and 150-bp paired-end sequencing by Illumina HiSeqX Ten with 30× average read depth was performed at Macrogen (Seoul, Korea).

### Analysis of rearranged sequences in WGS data

From the raw sequence data (FASTQ file), SAM files were prepared by the Burrows-Wheeler Aligner (BWA) (http://biobwa.sourceforge.net) using the UCSC Human Reference Genome hg19. BAM files were generated with SAMtools (http://samtools.sourceforge.net/). Quality control with FastQC (http://www.bioinformatics.babraham.ac.uk/projects/fastqc/) was performed by trimming data with a sequence quality score <30. The trimmed BAM file was sorted with SAMtools according to the leftmost coordinates and was indexed with SAMtools. The whole-genome data are summarized in Table S2.

Structural inter- and intrachromosomal rearrangements were detected with Manta^14^ in the tumor-normal analysis mode. The analyzed structural rearrangements were then visualized with the Integrative Genomics Viewer (http://software.broadinstitute.org/software/igv/); then, the rearranged sequences were constructed based on the whole-genome information for the rearrangements. From the comparison of the tumor and matched normal translocation results, the regions shown on both were excluded. The rearranged sites from the WGS are summarized in Table S3.

### Confirmation of the selected rearrangements in cancer DNAs

To amplify the rearranged sequences, PCR primers were designed with Primer3. PCR primers for long rearranged sequences (200–1000 bp) were designed for candidate rearranged sites from the WGS data (marked as long PCR in Table S4). After amplifying the DNAs from the paired tumor and normal samples, the rearranged sequences were confirmed by Sanger sequencing the amplified cancer-specific PCR products. After excluding the nonspecific amplifications, PCR primers for the short rearranged sequences at the confirmed rearranged sites were designed again (marked as short PCR in Table S4), and the specific rearranged sequences were confirmed again by PCR with short primers and with Sanger sequencing by employing DNAs from the paired tumor and normal samples. PCR was performed for each sequence under the following conditions: initial incubation at 95 °C for 10 min, followed by 45 cycles of 30 s each at 95 °C, 30 s at the annealing temperature for each primer pair, and 30 s at 72 °C; the reaction was performed in a mixture containing 1× PCR buffer II (Roche, Mannheim, Germany) with 1.5 mM MgCl_2_, 0.2 mM dNTPs, 10 pmol of each primer, and 10 ng of genomic DNA in a final volume of 20 μL. For some specific PCR amplifications, modifications were made, as indicated in Table S4. For the amplification controls, GAPDH primers (Table S4) were used. The amplified products were purified using the AxyPrep PCR Clean up kit (Axygen, Union City, CA) to remove leftover primers and were then sequenced with the forward or reverse primers used for PCR (Tables S4).

### Detection of rearranged sequences in plasma cell-free DNA

Cell-free DNA (cfDNA) from plasma was prepared using the QIAamp circulating nucleic acid kit (Qiagen, Hilden, Germany) according to the instruction manual, with an input plasma volume of 1 ml and an elution volume of 30 μL. PCR was performed under the same conditions as above, except that 2 μL of eluted cfDNA was used for each PCR. The PCR product amplified from each cfDNA sample was used for confirmation by Sanger sequencing.

To monitor the ctDNA levels in plasma, all available remnant plasma from the postoperative ctDNA-positive samples (GC4, GC8, GC9, GC14, GC15, GC17, and GC22) and plasmas from selected few postoperative ctDNA-negative samples (GC12, GC18, GC31, GC32, GC33, and GC34) were employed for quantitative PCR. Quantitative PCR was performed at one site for each sample, and the primer sequences are indicated in Table S5. Quantitative PCR was carried out according to the manufacturer’s protocol from FastStart Essential DNA Probes Master (Roche) by the LightCycler® 96 Real-Time PCR System (Roche) in a 25 µL reaction mixture consisting of 10 µL 2× FastStart Essential DNA Probes Master mix, 10 µL cfDNA (out of a total of 30 μL eluted cfDNA from 1 ml plasma, equivalent to 333 µL plasma) for both the personalized cancer-specific rearranged sequence and the reference gene, *GAPDH*, and primers (10 pmole each). To confirm the ctDNA negativity in the preoperative ctDNA negative samples (GC1, GC6, GC10, and GC12) by quantitative PCR, 25 µL cfDNA (equivalent to 833 µL plasma) and 5 µL cfDNA (equivalent to 167 µL plasma) were employed for the rearranged sequences and for the reference gene, *GAPDH*, respectively.

### Statistical analysis

To analyze preoperative ctDNA positivity and clinical factors including T stage, N stage, clinical stage, and Lauren classification, Fisher’s exact tests were used. To analyze the correlation between postoperative ctDNA positivity and clinical recurrence, Fisher’s exact tests were also used with postoperative ctDNA being considered positive when (1) any cancer-specific rearranged sequence was detected in any postoperative plasma sample within 12 months after surgery or (2) ctDNA-positive cases were detected only prior to clinical recurrence.

## Results

### LCM and DNA purification

**Fig. 1:**
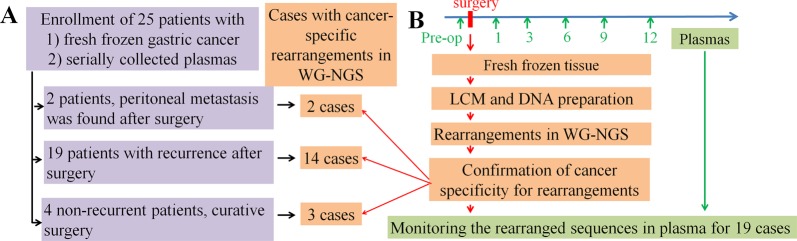
Study scheme. **a** Cases included in the present study. **b** Methodological procedure of the present study. DNA was prepared after laser-capture microdissection (LCM). Rearranged sequences were analyzed from WGS and were confirmed by PCR sequencing. The presence of ctDNA was monitored by PCR amplification of the personalized cancer-specific rearranged sequences in plasma samples

**Fig. 2:**
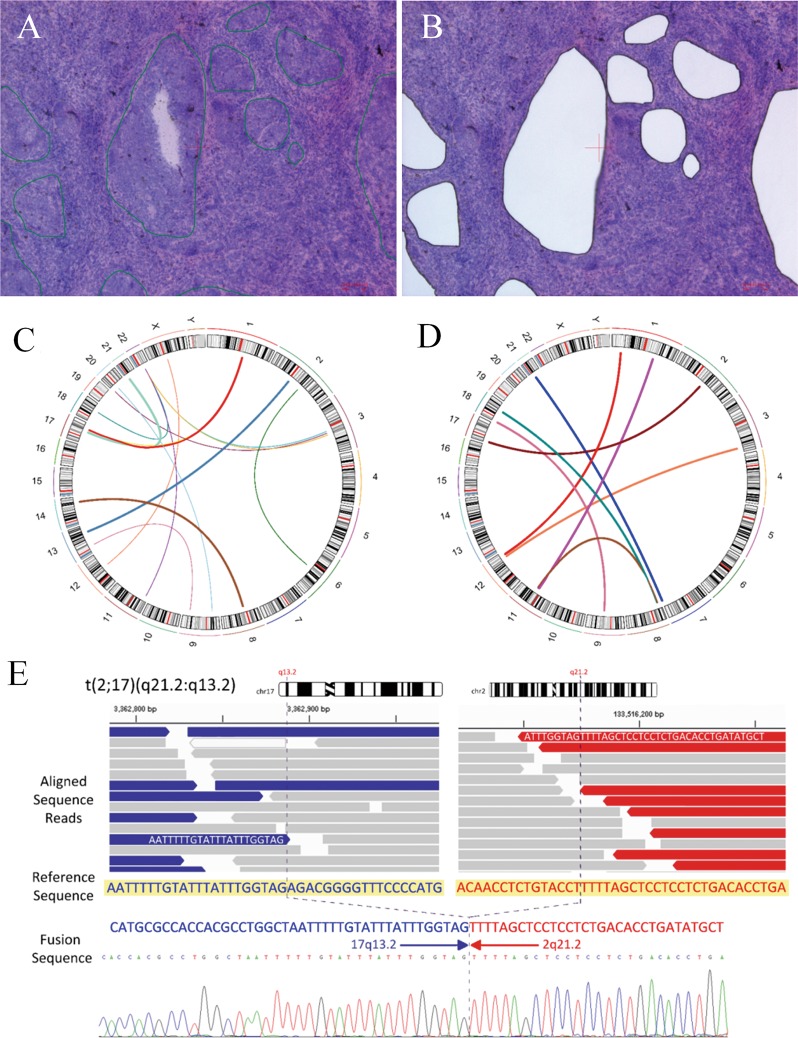
Identification of personalized cancer-specific rearrangements. **a** Cancer cells in primary tumor tissues before LCM. Cancer cell nests are marked with green lines. **b** Remnant normal cells and inflammatory cells after LCM. **c** Circos diagram of the rearrangements in GC17. The inter- and intrachromosomal arcs in the center indicate chromosomal rearrangements. **d** Circos diagram for GC32. **e** Analysis of the rearranged sequence marked in D for GC32 by Sanger sequencing

Among the 178 patients with available serial plasma samples up to 12 months after curative surgical resection (stage II, *N* = 69; stage III, *N* = 84; stage IV, *N* = 24; GIST, *N* = 1), all 21 recurrent patients (stage II, *N* = 2; stage III, *N* = 16; stage IV, *N* = 2; GIST, *N* = 1) and 4 nonrecurrent patients (stage II, *N* = 2; stage III, *N* = 2) with available fresh-frozen paired tumor and normal samples from the Tissue Bank of the National Cancer Center were selected (Fig. 1). Peritoneal seeding was diagnosed in two stage IV patients after surgical removal of the primary tumor. The clinical information of all patients is summarized in Table 1. LCM was performed on the fresh-frozen primary tumors to enrich the cancer cells (Fig. 2a, b), and the estimated cancer cell percentages after LCM were above 70%.

### Identification of personalized cancer-specific rearranged sequences

To identify personalized cancer-specific rearrangements that could serve as biomarkers, WGS was performed on DNAs isolated from 25 paired primary gastric cancer and normal gastric tissues. On average, 796 million DNA fragments were sequenced per tumor (range: 683–933 million), yielding a mean genome sequence coverage of 41.7-fold (range: 35.8–48.9) (Table S2). After analysis of the WGS data, the rearranged sequences specific to the tumor samples were identified (Fig. 2c–e, Table S3). In six cases, no personalized cancer-specific rearrangement was identified in the WGS data, and no further analysis was performed. PCR primers were designed for 141 sites from 19 out of 384 cancer-specific rearrangements identified in the WG-NGS data (Table S4). Some of the remaining 243 rearranged sites contained highly repetitive sequences, and the specific primer design was inadequate. Of the 141 primer pairs, cancer-specific amplification was observed at 84 sites (Table 1). With Sanger sequencing, personalized cancer-specific rearranged sequences were confirmed at 66 sites (Fig. 2e, Table 1). Therefore, the false-positive rate of identifying rearrangement by WG-NGS was 53.2% (75/141) in the present study.

**Fig. 3:**
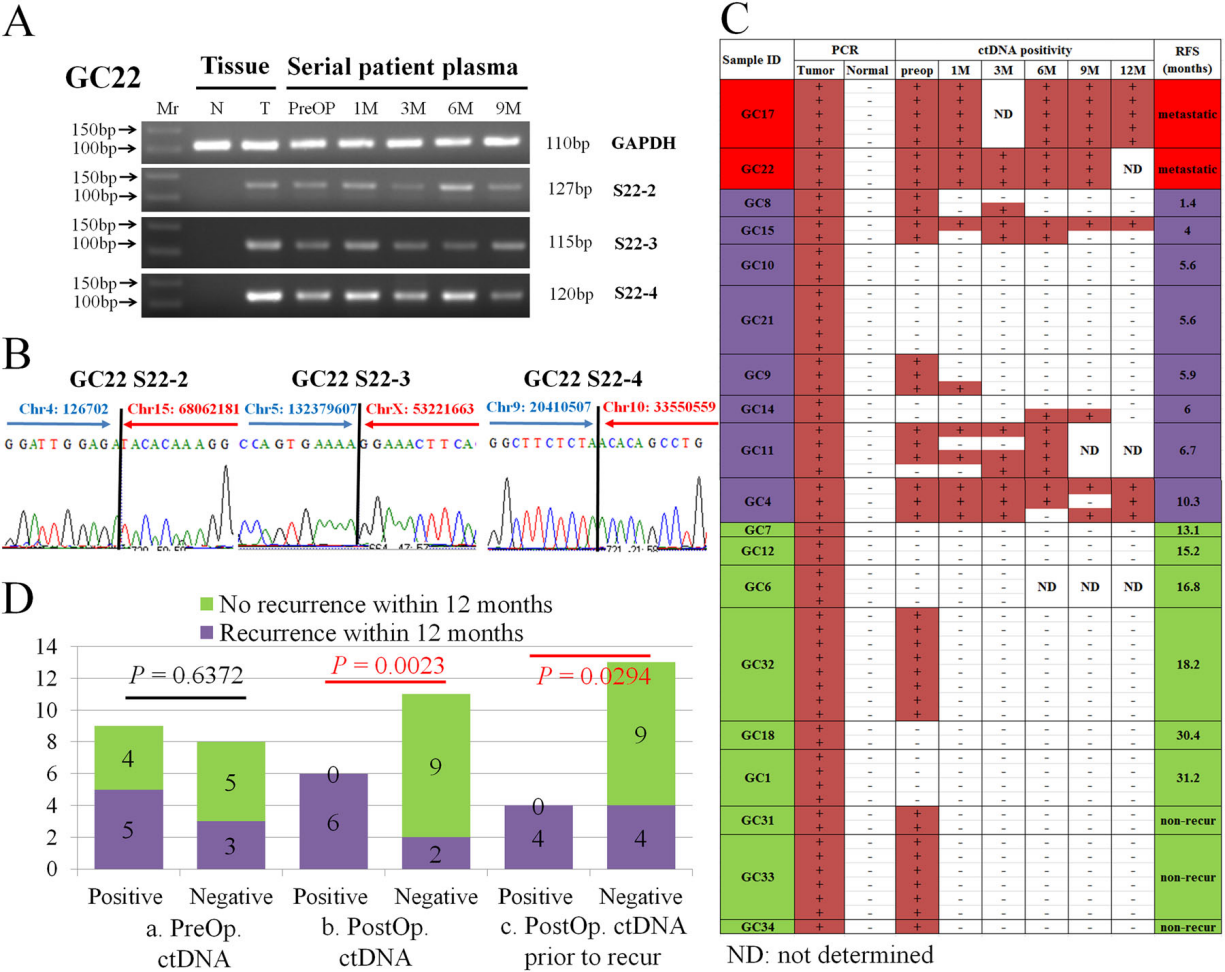
Monitoring of ctDNA in serially collected plasma samples. **a** Confirmation of the rearrangements by PCR. Rearranged sequences (S22-2, S22-3, and S22-4) were amplified in preoperative (PreOP) and serial postoperative plasma samples collected at 1–9 months (1 M–9 M) after surgery, along with normal (N) and tumor (T) tissue samples. Mr, molecular size markers. **b** Confirmation of three rearrangements (S22-2, S22-3, and S22-4) by Sanger sequencing. **c** ctDNA positivity in 19 gastric cancer patients. Each line indicates one personalized cancer-specific rearranged marker: + positive ctDNA; − negative ctDNA; RFS, relapse-free survival in months. Cases of metastasis are marked in red; cases of recurrence within 1 year of surgery are marked in purple; and cases of recurrence after 1 year or cases of no recurrence are marked in green. **d** Correlation of ctDNA positivity with recurrent events within 12 months postoperatively. (a) Correlation of preoperative ctDNA positivity with recurrent events. (b) Correlation of postoperative ctDNA positivity with recurrent events. (c) Correlation of postoperative ctDNA positivity prior to clinical recurrence with recurrent cancer

With the Sanger sequencing data, specific primers were designed again for short-length PCR products. With the designed short primer pairs, the rearranged sequences were confirmed by cancer-specific PCR and Sanger sequencing at 63 rearranged sites (Table S4, Figs. 3a, b), and these personalized cancer-specific short primers were used to monitor ctDNA in plasma samples^11^.

### Monitoring the presence of ctDNA in serial plasma samples

Circulating cell-free DNA was isolated from 107 plasma samples from 19 patients. Each personalized cancer-specific PCR was performed along with positive (tumor DNA) and negative (paired normal DNA) controls (Fig. 3a). To confirm the rearranged sequences, the amplified products were sequenced by the Sanger sequencing method (Fig. 3b).

In the preoperative plasma samples, ctDNA was positive in 11 patients, and the positivity rate of preoperative ctDNA in advanced gastric cancer patients was 58% (11/19) (*P* = 0.0587 by Fisher’s exact test, Table 1). In the analysis of preoperative ctDNA positivity and the clinical T stages (tumor size) of the gastric cancer patients, there was no significant correlation between the two factors (*P* = 0.3189), although the number of cases was quite low. None of the other clinical factors, including N stage, clinical stage, or Lauren classification, were significantly correlated with preoperative ctDNA positivity. ctDNA was detected in the postoperative plasma samples from eight patients, and the median lead time from ctDNA positivity to clinical recurrence after ctDNA detection was 4.05 months (Table 1). The two patients with clinical stage IV disease who was found to have positive peritoneal seeding after surgical resection showed positive ctDNA in the postoperative plasma. In seven patients, no ctDNA was detected in pre- or postoperative plasma samples, even with 3–5 different markers (Fig. 3c).

In the analysis of the correlation between postoperative ctDNA positivity and clinical recurrence, the presence of postoperative ctDNA at any time within 12 months of the surgical resection was significantly correlated with cancer recurrence within 12 months of the surgical resection (*P* = 0.0023, Fig. 3Db); in contrast, no significant correlation was found between cancer recurrence within 12 months of the surgical resection and preoperative ctDNA positivity (*P* = 0.6372, Fig. 3Da). For this analysis, ctDNA was considered positive when any cancer-specific rearranged sequence was detected in any plasma sample. However, this correlation might not be properly indicative of the usefulness of ctDNA monitoring because ctDNA-positive samples detected after clinical recurrence were also included in the positive correlation. To remove this error, only ctDNA-positive sampled detected prior to clinical recurrence were analyzed as postoperative ctDNA-positive cases, and the results once again indicated a significant correlation between ctDNA positivity prior to clinical recurrence and cancer recurrence within 12 months of curative surgical resection (*P* = 0.0294, Fig. 3Dc), suggesting that ctDNA positivity can be an indicator of future clinical recurrence.

A statistical analysis of the correlation between adjuvant chemotherapy (Table S1) and postoperative ctDNA negativity revealed an insignificant relationship due to the limited number of samples. However, all three nonrecurrent preoperative ctDNA-positive patients who received adjuvant chemotherapy were negative for postoperative ctDNA, in contrast to both preoperative ctDNA-positive patients without adjuvant chemotherapy who were positive for postoperative ctDNA.

**Fig. 4:**
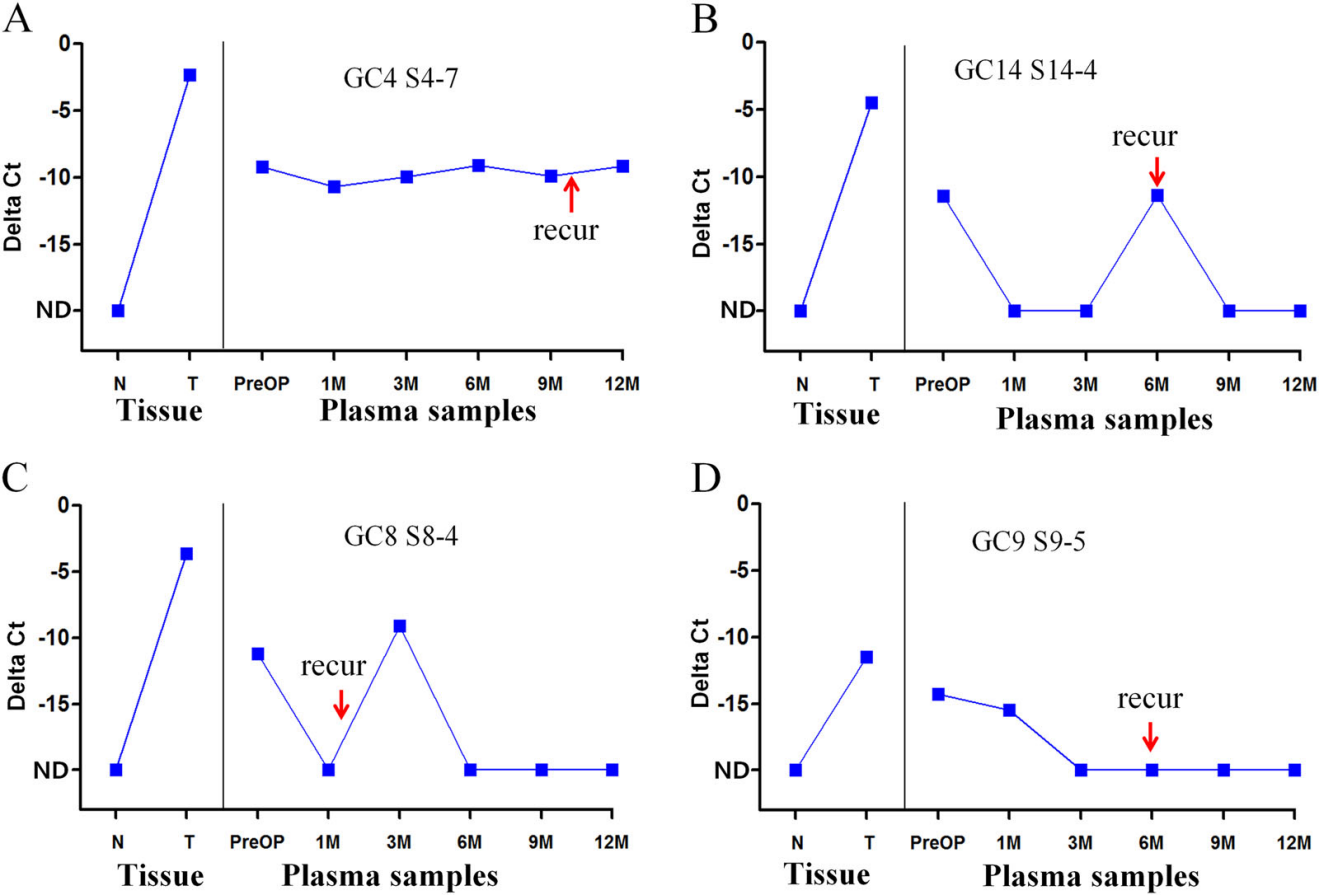
Quantitative measurements of the ctDNA levels in serial plasma samples from gastric cancer patients. **a** ctDNA levels in a cancer patient, GC4. **b** ctDNA levels in GC14. C. ctDNA levels in GC8. D. ctDNA levels in GC9. *GAPDH*, amplification control. *X*-axis, DNAs from normal (N) and cancer (T) tissues and from preoperative (PreOP) and postoperative (PostOp) plasma samples at 1, 3, 6, 9, and 12 months after surgery. *Y*-axis, delta Ct (the difference in Ct values between the marker and *GAPDH*). The arrows indicate the time of clinical recurrence after surgery. *ND*, nondetectable

To quantitatively measure the ctDNA in plasma, quantitative PCR was performed for 13 samples. Amplification was confirmed in 96.1% (74/77) of the plasma samples in which the presence of ctDNA was tested by PCR and Sanger sequencing (Fig. 4, Table S6). The detection of ctDNA can help to predict clinical recurrence, as shown in Fig. 4a; however, for the cases shown in Fig. 4b, c, this measurement would not be helpful because the date of detection is similar to or later than the date of clinical recurrence. In one case (Fig. 4d), ctDNA was detected 1 month after surgery but not after clinical recurrence. To evaluate if there were more ctDNA-positive cases, quantitative PCR was performed with the preoperative blood samples from five ctDNA-negative patients with sample amounts equivalent to 333 µL (for GC12 and GC18) or 833 µL (for GC1, GC6, GC10, and GC12) of plasma, but all were negative (Table S7).

In our quantitative results, the ctDNA levels in most of the pre- and postoperative plasma samples were at the lower limit of quantitative PCR detection (mean Ct value: 37.8), which limits the quantitative value of the ctDNA. The difference in the ctDNA levels between the preoperative and postoperative plasma was not large (2–4 cycles) relative to the expected difference based on the dramatic reduction in tumor size after curative surgical treatment.

We performed droplet digital PCR (ddPCR) for two markers in the GC4 sample, employing 10 µL cfDNA (equivalent to 333 µL plasma) to ensure the positive identification of ctDNA in postoperative plasma. In all of the postoperative plasma samples for the two markers, ctDNA was positive by ddPCR, which corresponds to our results from quantitative PCR with the same amount (10 µL) of cfDNA, as shown in Table S8.

## Discussion

By analyzing the ctDNA levels in postoperative blood with personalized cancer-specific rearrangements instead of mutations, we confirmed the presence of ctDNA at a median lead time of 4.05 months and found that postoperative ctDNA positivity prior to clinical recurrence was significantly correlated with cancer recurrence within 12 months of radical surgery (*P* = 0.029). As such, our study confirmed the clinical usefulness of ctDNA monitoring for cancer recurrence in gastric cancer patients after curative surgical resection.

Although ctDNA has been detected in blood samples obtained from cancer patients, the usefulness of ctDNA for the detection of early recurrence after curative surgical resection has been in question due to the possibility of low levels of ctDNA shedding from microscopically remnant or recurrent cancer cells when considering the general correlation between the tumor burden and the ctDNA level^15^. Two studies on ctDNA in blood for monitoring recurrence in breast cancer^7^ and colon cancer^8^ suggested the possibility of clinical applications for mutation monitoring. The employment of mutations for serial ctDNA monitoring, however, can suffer from a high rate of inconsistency due to false positivity or negativity or technical NGS problems in the detection of mutations, especially for low-allele-frequency mutants^16^. The proportion of ctDNA in the blood is extremely low, and thus NGS methods must be effective in detecting mutant allelic frequencies as low as 0.1%^17^, which might lead to inconsistencies in ctDNA detection by NGS. A comparative study of mutations in primary tumors and ctDNA from the blood of advanced lung cancer patients also indicated that there would be inconsistencies when mutation calls obtained from NGS are employed to monitor ctDNA: the concordance rate was only 50.4%, even in the blood from cancer patients who had not undergone surgical removal of their primary tumors^18^. To alleviate the problem of inconsistency in NGS, personalized cancer-specific rearrangements have been employed for the detection of ovarian cancer recurrence^19^, with the resultant data confirming the presence of ctDNA in the postoperative blood; however, the clinical usefulness of ctDNA was not analyzed in that study. The present study, which employed cancer-specific rearrangements, established the clinical usefulness of ctDNA monitoring for cancer recurrence: the presence of ctDNA was confirmed at a median lead time of ∼4 months, which demonstrated the significant association between ctDNA presence in the blood prior to clinical recurrence and cancer recurrence within 12 months of the curative surgical resection. Therefore, our study can be considered to advocate for the utility of ctDNA monitoring for cancer recurrence after curative surgical resection.

Although previous studies have shown that ctDNA can be an excellent screening method for cancer recurrence, significant fractions of the recurrent cancer patients in those studies showed ctDNA negativity in their postoperative blood^3,7^. The main suggested factors behind those results were tumor heterogeneity and the relative paucity of remnant cancer cells after curative resection. Inconsistent postoperative ctDNA positivity in each rearranged sequence of some samples in the present study might indicate the possible heterogeneity of cancer cells, which would necessitate the employment of several rearranged markers for one sample to increase the postoperative ctDNA positivity. In addition, ctDNA non-shedders that do not have detectable ctDNA in preoperative blood may be one of the main reasons behind the ctDNA-negativity in recurrent postoperative blood because many patients in the present study had no ctDNA in their preoperative blood, although all recruited patients had T3 or T4 stage disease. Moreover, most of the preoperative ctDNA-negative patients (7/8) and recurrent ctDNA non-shedders (4/5) remained ctDNA-negative according to the postoperative blood. Consistent with this finding, preoperative ctDNA-negative cases and ctDNA non-shedders have already been reported^6^. Therefore, ctDNA non-shedders might be an important reason for ctDNA negativity in recurrent cases. The inclusion of only preoperative ctDNA-positive cases or ctDNA shedders for ctDNA monitoring might, accordingly, improve the cost effectiveness for the early detection of cancer recurrence after curative surgical treatment.

In the present study on the serial monitoring of ctDNA in postoperative blood, several issues arose. First, the presence of ctDNA in serial postoperative blood was not consistently positive during the follow-up periods. For example, in one patient, the presence of ctDNA was positive at 1 month following surgical resection but became negative until clinical recurrence, which suggests that ctDNA levels during the follow-up periods might continually change with ctDNA dynamics. Therefore, the meaning of ctDNA positivity in the short term, as this measure relates to cancer recurrence risk, might be difficult to determine. At the very least, more frequent monitoring of ctDNA could increase the chances of correctly identifying patients likely to have recurrent disease or could help to determine the risk for cancer recurrence. The second issue that arose in this study with respect to the serial monitoring of ctDNA in postoperative blood was the fact that the level of postoperative ctDNA was not remarkably different from the corresponding preoperative level, although a large decrease in tumor burden was expected after the curative surgical removal of the primary cancer. Previous studies employing mutations for ctDNA monitoring have also reported cases of small changes in ctDNA levels between the pre- and postoperative blood^7,20^, suggesting that factors other than tumor size might also be important for determining ctDNA levels. Although ctDNA levels have been reported to be correlated with tumor size^17^, there were no significant correlations in the present study between pre- or postoperative ctDNA positivity and T stage (tumor size), which supports the belief that inherent biological or dynamic tumor factors determine ctDNA levels. Therefore, issues such as the presence of ctDNA in the short-term follow-up and ctDNA dynamics independent of tumor size could be considered to interfere with the accurate prediction of cancer recurrence by ctDNA monitoring.

In the present study, personalized cancer-specific rearrangements were employed for monitoring ctDNA in postoperative blood samples obtained from cancer patients. We expected that the monitoring of rearrangements in postoperative blood would be sensitive, simple, and rapid for more frequent monitoring of ctDNA. Although employing mutations has dramatically increased the sensitivity^12,13,21^, the serial monitoring of mutations in postoperative blood by NGS or droplet digital PCR would take more time and cost more than simple PCR. However, the time and cost burdens of obtaining information on cancer-specific rearrangements by WGS is high. In particular, high proportions of rearrangements detected in WGS analysis are negative with PCR confirmation or PCR sequencing. Furthermore, in the present study, WGS failed to find any cancer-specific rearrangements in 6 out of 25 samples, adding to the difficulty of employing rearrangements for ctDNA monitoring. Therefore, to employ personalized cancer-specific rearrangements in the monitoring of ctDNA, more time- and cost-effective screening methods are necessary.

The present study has several limitations. This study was performed retrospectively; the plasma samples were collected up to 12 months after the curative surgical resection, and the available recurrence samples were selected preferentially, which are both conditions that can incur bias. A prospective study with more extensive serial collections of plasma samples up to cancer recurrence would yield more objective information on ctDNA monitoring for cancer recurrence. Additionally, the present study employed only limited amounts of plasma, ∼1 ml in most cases and only ∼67 µL of plasma per PCR to monitor ctDNA, because several markers had to be simultaneously evaluated. The use of larger volumes of plasma for ctDNA monitoring would increase sensitivity.

In conclusion, we demonstrated the usefulness of ctDNA monitoring with personalized cancer-specific rearranged sequences to detect gastric cancer recurrence, confirmed the presence of ctDNA at a median lead time of 4.05 months, and showed the significant correlation of ctDNA positivity with clinical recurrence. Our results also raise the following important issues that could limit the usefulness of ctDNA monitoring: (1) ctDNA non-shedders without any detectable preoperative ctDNA, most of whom remain ctDNA non-shedders even after cancer recurrence; and (2) inconsistent postoperative ctDNA positivity in ctDNA shedders. In consideration of our overall results, ctDNA monitoring for cancer recurrence certainly warrants future prospective studies on the clinical utility of this method, but the limitations due to ctDNA dynamics during the pre- and postoperative periods should be considered when designing prospective studies.

## Supplementary information

Table S1

Table S2

Table S3

Table S4

Table S5

Table S6

Table S7

Table S8

Figure S1
