# Supplementary material for: Monitoring circulating tumor DNA by analyzing personalized cancer-specific rearrangements to detect recurrence in gastric cancer
Source: Exp Mol Med. 2019 Aug 8;51(8):93. doi: 10.1038/s12276-019-0292-5 (PMC6802636; doi:10.1038/s12276-019-0292-5)
Supplement: Supplementary file 1 — Table S1 [file 12276_2019_292_MOESM1_ESM.docx]

Table S1. Clinical information for gastric cancer patients accrued in the present study

| **ID** | **Lauren** | **Histology*** | **Recurrent sites** | **Adjuvant Chemotherapy** |
| --- | --- | --- | --- | --- |
| GC1 | Intestinal | Mod | Celiac axis LN | No |
| GC4 | Intestinal | Poor + Mucin | Pancreas, Aorto-caval LN | Yes |
| GC6 | Intestinal | Mod + Mucin | GJ Anastomosis , peritoneum, pleural | No |
| GC7 | Intestinal | Mod | Duodenal stump, Porto-caval LN | No |
| GC8 | Intestinal | Poor + Mucin | Peritoneum | Yes (palliative) |
| GC9 |  | GIST | Liver, peritoneum | No |
| GC10 | Intestinal | Mod + Neuro | Liver | No |
| GC11 | Intestinal | Mod | Liver | No |
| GC12 | Diffuse | Poor | Abdominal wall, mesentery | No |
| GC14 | Intestinal | Poor | Aortico-caval and porto-caval LN | Yes |
| GC15 | Diffuse | Poor | Para-aortic LN | Yes |
| GC17 | Mixed | Poor + Mod | Bone, Peritoneum | Yes (palliative) |
| GC18 | Diffuse | Signet | Colon | No |
| GC21 | Intestinal | Mucin | Peritoneum | Yes |
| GC22 | Mixed | Poor | Duodenal stump, para-arotic and thorax LN, peritoneum | Yes (palliative) |
| GC31 | Intestinal | Mod | - | Yes |
| GC32 | Diffuse | Poor | Gastrojejunostomy site | Yes |
| GC33 | Intestinal | Poor | - | Yes |
| GC34 | Diffuse | Poor + Mucin | - | Yes |
| CG35 | Diffuse | Poor | - | Yes |
| GC2 | Diffuse | Signet | Ovary | No |
| GC3 | Intestinal | Mod | Peritoneum | No |
| GC5 | Diffuse | Poor | LN (celiac, SMA) Peritoneum | Yes |
| GC13 | Intestinal | Mod | Abdominal wall, Peritoneum | No |
| GC23 | Intestinal | Poor | Liver | Yes |

*Mod, moderate differentiated adenocarcinoma; Poor, poorly differentiated adenocarcinoma; Mucin, mucinous adenocarcinoma; Signet, signet ring cell carcinoma; GIST, gastrointestinal stromal tumor
