# Supplementary material for: Monitoring circulating tumor DNA by analyzing personalized cancer-specific rearrangements to detect recurrence in gastric cancer
Source: Exp Mol Med. 2019 Aug 8;51(8):93. doi: 10.1038/s12276-019-0292-5 (PMC6802636; doi:10.1038/s12276-019-0292-5)
Supplement: Supplementary file 2 — Table S2 [file 12276_2019_292_MOESM2_ESM.docx]

Table S2. The summary of whole genome shotgun data

| **Samples*** | **Sequencing reads**** | **Read length (bp)** | **Total yield (Mbp)** | **Throughput mean depth (X)** | **De-duplicated reads** | **De-duplicated reads % (out of total reads)** | **Mappable reads** | **Mappable reads % (out of De-duplicated reads)** | **Mappable yield (Mbp)** | **Mappable mean depth (X)** | **% ≥ 20X coverage** | **% ≥30X coverage** |
| --- | --- | --- | --- | --- | --- | --- | --- | --- | --- | --- | --- | --- |
| C1 | 709,303,590 | 150 | 106,395 | 37.2 | 621,425,520 | 87.6 | 596,493,644 | 96.0 | 89,474 | 31.3 | 87.9 | 59.3 |
| C2 | 781,193,946 | 150 | 117,179 | 41.0 | 668,308,648 | 85.5 | 639,376,982 | 95.7 | 95,906 | 33.5 | 95.7 | 80.2 |
| C3 | 712,376,406 | 150 | 106,856 | 37.4 | 626,052,682 | 87.9 | 598,921,057 | 95.7 | 89,838 | 31.4 | 91.7 | 69.3 |
| C4 | 698,425,098 | 150 | 104,763 | 36.6 | 605,139,392 | 86.6 | 577,735,166 | 95.5 | 86,660 | 30.3 | 87.8 | 55.9 |
| C5 | 791,615,928 | 150 | 118,742 | 41.5 | 680,288,418 | 85.9 | 650,897,946 | 95.7 | 97,634 | 34.2 | 95.4 | 77.7 |
| C6 | 708,000,838 | 150 | 106,200 | 37.2 | 613,134,292 | 86.6 | 585,320,581 | 95.5 | 87,798 | 30.7 | 91.2 | 63.7 |
| C7 | 812,888,522 | 150 | 121,933 | 42.7 | 693,087,484 | 85.3 | 660,310,511 | 95.3 | 99,046 | 34.6 | 93.9 | 80.3 |
| C8 | 896,810,136 | 150 | 134,521 | 47.1 | 736,514,414 | 82.1 | 696,906,858 | 94.6 | 104,536 | 36.6 | 95.3 | 85.3 |
| C9 | 844,658,256 | 150 | 126,698 | 44.3 | 705,211,798 | 83.5 | 670,322,267 | 95.1 | 100,548 | 35.2 | 91.3 | 76.3 |
| C10 | 790,685,234 | 150 | 118,602 | 41.5 | 678,658,800 | 85.8 | 648,873,581 | 95.6 | 97,331 | 34.0 | 94.1 | 74.1 |
| C11 | 793,476,142 | 150 | 119,021 | 41.6 | 728,996,018 | 91.9 | 703,929,460 | 96.6 | 105,589 | 36.9 | 92.5 | 68.7 |
| C12 | 875,516,716 | 150 | 131,327 | 45.9 | 796,699,078 | 91.0 | 767,490,239 | 96.3 | 115,123 | 40.3 | 95.0 | 81.3 |
| C13 | 833,771,924 | 150 | 125,065 | 43.7 | 753,325,656 | 90.4 | 720,937,639 | 95.7 | 108,140 | 37.8 | 97.2 | 83.9 |
| C14 | 743,913,492 | 150 | 111,587 | 39.0 | 674,596,142 | 90.7 | 642,903,243 | 95.3 | 96,435 | 33.7 | 93.5 | 69.9 |
| C15 | 774,265,852 | 150 | 116,139 | 40.6 | 706,541,954 | 91.3 | 671,262,631 | 95.0 | 100,689 | 35.2 | 94.7 | 73.3 |
| C17 | 818,329,106 | 150 | 122,749 | 42.9 | 744,193,362 | 90.9 | 711,771,309 | 95.6 | 106,765 | 37.3 | 95.1 | 78.7 |
| C18 | 832,615,954 | 150 | 124,892 | 43.7 | 757,690,626 | 91.0 | 721,345,196 | 95.2 | 108,201 | 37.9 | 96.2 | 86.2 |
| C21 | 932,670,882 | 150 | 139,900 | 48.9 | 849,929,324 | 91.1 | 830,675,054 | 97.7 | 124,601 | 43.6 | 85.1 | 69.8 |
| C22 | 792,898,934 | 150 | 118,934 | 41.6 | 729,640,526 | 92.0 | 689,123,721 | 94.4 | 103,368 | 36.2 | 89.0 | 63.7 |
| C23 | 896,658,266 | 150 | 134,498 | 47.0 | 820,943,624 | 91.6 | 786,588,769 | 95.8 | 117,988 | 41.3 | 79.5 | 59.9 |
| C31 | 888,793,264 | 150 | 133,318 | 47 | 795,751,050 | 90 | 754,591,355 | 95 | 113,188 | 40 | 96 | 80 |
| C32 | 850,050,766 | 150 | 127,507 | 45 | 757,429,632 | 89 | 720,588,360 | 95 | 108,088 | 38 | 89 | 73 |
| C33 | 849,173,488 | 150 | 127,376 | 45 | 751,909,772 | 89 | 710,958,849 | 95 | 106,643 | 37 | 95 | 84 |
| C34 | 761,028,374 | 150 | 114,154 | 40 | 654,636,996 | 86 | 611,376,192 | 93 | 91,706 | 32 | 94 | 76 |
| C35 | 740,272,224 | 150 | 111,040 | 39 | 670,048,280 | 91 | 628,028,992 | 94 | 94,204 | 33 | 94 | 74 |
| N1 | 755,041,068 | 150 | 113,256 | 39.6 | 629,405,906 | 83.4 | 598,474,285 | 95.1 | 89,771 | 31.4 | 93.3 | 74.7 |
| N2 | 753,760,804 | 150 | 113,064 | 39.6 | 618,419,722 | 82.0 | 588,922,198 | 95.2 | 88,338 | 30.9 | 95.6 | 76.1 |
| N3 | 890,317,982 | 150 | 133,547 | 46.7 | 719,723,830 | 80.8 | 687,210,434 | 95.5 | 103,081 | 36.1 | 95.9 | 87.3 |
| N4 | 769,863,394 | 150 | 115,479 | 40.4 | 633,742,190 | 82.3 | 602,626,990 | 95.1 | 90,394 | 31.6 | 93.8 | 76.4 |
| N5 | 756,558,900 | 150 | 113,483 | 39.7 | 626,999,518 | 82.9 | 596,709,074 | 95.2 | 89,506 | 31.3 | 95.0 | 75.7 |
| N6 | 794,694,578 | 150 | 119,204 | 41.7 | 650,189,616 | 81.8 | 619,773,551 | 95.3 | 92,966 | 32.5 | 94.5 | 80.1 |
| N7 | 780,488,730 | 150 | 117,073 | 41.0 | 634,288,872 | 81.3 | 600,064,513 | 94.6 | 90,009 | 31.5 | 94.0 | 78.1 |
| N8 | 760,362,986 | 150 | 114,054 | 39.9 | 636,638,606 | 83.7 | 602,714,906 | 94.7 | 90,407 | 31.6 | 93.7 | 76.1 |
| N9 | 725,814,732 | 150 | 108,872 | 38.1 | 638,267,818 | 87.9 | 608,036,087 | 95.3 | 91,205 | 31.9 | 92.3 | 71.0 |
| N10 | 717,860,220 | 150 | 107,679 | 37.7 | 636,986,404 | 88.7 | 611,023,215 | 95.9 | 91,653 | 32.1 | 92.3 | 71.1 |
| N11 | 832,545,506 | 150 | 124,881 | 43.7 | 763,407,340 | 91.7 | 712,859,717 | 93.4 | 106,928 | 37.4 | 95.3 | 83.9 |
| N12 | 682,868,058 | 150 | 102,430 | 35.8 | 636,499,610 | 93.2 | 591,066,010 | 92.9 | 88,659 | 31.0 | 91.7 | 60.8 |
| N13 | 755,065,092 | 150 | 113,259 | 39.6 | 699,560,412 | 92.6 | 656,750,764 | 93.9 | 98,512 | 34.5 | 96.5 | 76.7 |
| N14 | 759,208,722 | 150 | 113,881 | 40 | 697512430 | 92 | 650696589 | 93 | 97604 | 34 | 94 | 75 |
| N15 | 743,341,192 | 150 | 111,501 | 39 | 690719758 | 93 | 642778700 | 93 | 96416 | 34 | 94 | 73 |
| N17 | 804,629,024 | 150 | 120,694 | 42 | 740433446 | 92 | 698284822 | 94 | 104742 | 37 | 95 | 83 |
| N18 | 884,177,552 | 150 | 132,626 | 46 | 807540784 | 91 | 756408858 | 94 | 113461 | 40 | 96 | 88 |
| N21 | 797,243,836 | 150 | 119,586 | 42 | 738823662 | 93 | 696666129 | 94 | 104499 | 37 | 95 | 82 |
| N22 | 737,750,022 | 150 | 110,662 | 39 | 682215916 | 93 | 641215766 | 94 | 96182 | 34 | 94 | 74 |
| N23 | 757,958,182 | 150 | 113,693 | 40 | 698074374 | 92 | 656898693 | 94 | 98534 | 35 | 94 | 76 |
| 31N | 923,352,638 | 150 | 138,502 | 49 | 855,038,666 | 93 | 807,365,803 | 94 | 121,104 | 42 | 97 | 91 |
| 32N | 826,421,956 | 150 | 123,963 | 43 | 762,635,160 | 92 | 720,791,758 | 95 | 108,118 | 38 | 96 | 86 |
| 33N | 810,046,520 | 150 | 121,506 | 43 | 739,885,782 | 91 | 699,652,296 | 95 | 104,947 | 37 | 96 | 84 |
| 34N | 814,124,764 | 150 | 122,118 | 43 | 757,778,802 | 93 | 716,601,822 | 95 | 107,490 | 38 | 95 | 84 |
| 35N | 819,133,546 | 150 | 122,870 | 43 | 757,192,374 | 92 | 708,490,893 | 94 | 106,273 | 37 | 96 | 84 |

*The tumor and matched normal genomes are discriminated with the use of 'C' and 'N', respectively.

**The mean and median coverage as well as the % of bases (>= 20 reads) were calculated onto the targeted regions.
