# Supplementary material for: Monitoring circulating tumor DNA by analyzing personalized cancer-specific rearrangements to detect recurrence in gastric cancer
Source: Exp Mol Med. 2019 Aug 8;51(8):93. doi: 10.1038/s12276-019-0292-5 (PMC6802636; doi:10.1038/s12276-019-0292-5)
Supplement: Supplementary file 5 — Table S5 [file 12276_2019_292_MOESM5_ESM.docx]

Table S5. Primer information for quantitative PCR.

| **No.** | **Primer ID** | **Primer sequences*** | **Annealing temperature (℃)** | **PCR product** |
| --- | --- | --- | --- | --- |
|  |  |  |  |  |
| 1 | GC S4-7 | F: 5'-ATGAGGCACTCCAAGCAAAG-3' | 55 | 107bp |
|  |  | R: 5'-TGGGAGAGAAAGGAAGGTTTT-3' |  |  |
|  |  | Probe: 5'-CAGCAGCAAGAATGCAAAAA-3' |  |  |
| 2 | GC S8-4 | F: 5'-AGCGTTCCATCACAGAATGA-3' | 55 | 130bp |
|  |  | R: 5'-CATTCCAGGCAACCAAAAAC-3' |  |  |
|  |  | Probe: 5'-ACCGCCTTTGCAAAATTATG-3' |  |  |
| 3 | GC S9-5 | F: 5'-TCCAGGTAGACGTGTCAAATAAA-3' | 55 | 120bp |
|  |  | R: 5'-TCCAGGTAGACGTGTCAAATAAA-3' |  |  |
|  |  | Probe: 5'-TGAAGTTCAAAACTAAGGTAAATTTGG-3' |  |  |
| 4 | GC S12-2 | F: 5’- CCCAAGTAGCTGGGAAAACA -3’ | 55 | 126bp |
|  |  | R: 5’- CCTCAGATGCACGTTCCA -3’ |  |  |
|  |  | Probe: 5'-ACGACACCCGGCTAATTTTT-3' |  |  |
| 5 | GC S14-4 | F: 5’- TCTAAGTAGTTTTTACCCATCCAAA -3’ | 52 | 106bp |
|  |  | R: 5’- ATGGGTCATCAAACAACTACAAAAG -3’ |  |  |
|  |  | Probe: 5'-TTGCCCAAGATCAGGATTTG-3' |  |  |
| 6 | GC SKN15-2 | F: 5’- TGGCTAAGTGGAGAGAAATGG -3’ | 55 | 121bp |
|  |  | R: 5’- TGGCTAAGTGGAGAGAAATGG -3’ |  |  |
|  |  | Probe: 5'-TGAGGTTTTGATATTTCACGTGA-3' |  |  |
| 7 | GC S17-1 | F: 5’- TGGTTCAGTTTCCGTATCTGT -3’ | 55 | 110bp |
|  |  | R: 5’- GAAGTGGGTTCTTCTAATCAAGC -3’ |  |  |
|  |  | Probe: 5'-GATCTGAATTGTGTCATTCATTCA-3' |  |  |
| 8 | GC SKN18-2 | F: 5’- GGTCTCTTTGTATATGACCTTCTCC -3’ | 55 | 130bp |
|  |  | R: 5’- TCCTTCCTTCTGGCAATAGAA -3’ |  |  |
|  |  | Probe: 5'-TGGAGGTGGAGTTGTGTTCA-3' |  |  |
| 9 | GC S22-3 | F: 5’- GGGTGGAGTTGGAACGTTAG -3’ | 55 | 115bp |
|  |  | R: 5’- AAAAACTGTGAGCACGGCTA -3’ |  |  |
|  |  | Probe: 5'-GGCTAGGTGAGGAGTGTTGG-3' |  |  |
| 10 | GC31 S5 | F: 5’- GCCAGTAATTGGGTATATTTTGG -3’ | 55 | 127bp |
|  |  | R: 5’- TTGGGAAACTAATGCAGGAAA -3’ |  |  |
|  |  | Probe: 5'-TTCACTAAGCATGTATGTGGAAA-3' |  |  |
| 11 | GC32 S2 | F: 5’- TGGTGGCATACACCTATTGC -3’ | 55 | 126bp |
|  |  | R: 5’- GAAGACAAAACCCACGGTTC -3’ |  |  |
|  |  | Probe: 5'-GTGAGAGGATTGCTTGAGCC-3' |  |  |
| 12 | GC33 S3 | F: 5’- TCAGGAGGAATTGGAGCCTA -3’ | 55 | 124bp |
|  |  | R: 5’- AGCTGGAATGGGTGATAAGG -3’ |  |  |
|  |  | Probe: 5'-AGAGGATGAAGGGCGAGAAG-3' |  |  |
| 13 | GC34 S2 | F: 5’- TCGACCTACTGCATGTCCTTT -3’ | 55 | 104bp |
|  |  | R: 5’- TGACAAAGGGCTAATATCCAGA -3’ |  |  |
|  |  | Probe: 5'-TCATCAATGAAAATGGGGGT-3' |  |  |
|  | GAPDH | F: 5'-TGCCTTCTTGCCTCTTGTCT-3' | 55 | 110bp |
|  |  | R: 5'-AATGAAGGGGTCATTGATGG-3' |  |  |
|  |  | Probe: 5'-TCACCAGGGCTGCTTTTAAC-3' |  |  |

*Primers for forward (F), reverse (R), and probe (Probe) sequences.
