# Supplementary material for: Monitoring circulating tumor DNA by analyzing personalized cancer-specific rearrangements to detect recurrence in gastric cancer
Source: Exp Mol Med. 2019 Aug 8;51(8):93. doi: 10.1038/s12276-019-0292-5 (PMC6802636; doi:10.1038/s12276-019-0292-5)
Supplement: Supplementary file 6 — Table S6 [file 12276_2019_292_MOESM6_ESM.docx]

Table S6. Estimation of relative level of ctDNA by quantitative PCR.

| **Sample ID** | **Tissue (Ct)** | |  | **ctDNA (Ct)** | | | | | | |
| --- | --- | --- | --- | --- | --- | --- | --- | --- | --- | --- |
|  | **Normal** | **Tumor** |  | **PreOP** |  | **PostOP** | | | | |
|  |  |  |  |  |  | **1M** | **3M** | **6M** | **9M** | **12M** |
| GC4 | - | 27.91 |  | 35.25 |  | 36.15 | 36.65 | 36.18 | 37.96 | 36.95 |
| GC8 | - | 28.22 |  | 38.62 |  | - | 37.56 | - | - | - |
| GC9 | - | 37.11 |  | 38.94 |  | 40.98 | - | - | - | - |
| GC12 | - | 26.16 |  | - |  | - | - | - | - | - |
| GC14 | - | 32.54 |  | 39.44 |  | - | - | 39.35 | N.M | - |
| GC15 | - | 30.90 |  | 37.57 |  | - | N.M | N.M | - | - |
| GC17 | - | 32.02 |  | 38.94 |  | 39.71 | N.D | 40.05 | 35.88 | 38.58 |
| GC18 | - | 26.00 |  | - |  | - | - | - | - | - |
| GC22 | - | 26.87 |  | 37.89 |  | 37.70 | 37.59 | 38.25 | 37.87 | N.D |
| GC31 | - | 26.46 |  | 35.25 |  | - | - | - | - | - |
| GC32 | - | 25.56 |  | 36.06 |  | - | - | - | - | - |
| GC33 | - | 28.39 |  | 37.17 |  | - | - | - | - | - |
| GC34 | - | 30.73 |  | 40.28 |  | - | - | - | - | - |

Ct, threshold cycles; -, not detected; N.M, not matched; N.D, not determined.
