# Supplementary material for: Monitoring circulating tumor DNA by analyzing personalized cancer-specific rearrangements to detect recurrence in gastric cancer
Source: Exp Mol Med. 2019 Aug 8;51(8):93. doi: 10.1038/s12276-019-0292-5 (PMC6802636; doi:10.1038/s12276-019-0292-5)
Supplement: Supplementary file 7 — Table S7 [file 12276_2019_292_MOESM7_ESM.docx]

Table S7. Estimation of the level of pre-operative ctDNA by quantitative PCR in 5 pre-operative ctDNA-negative cases.

| Sample ID | *GAPDH* (Ct) | | |  | Target (Ct) | | |
| --- | --- | --- | --- | --- | --- | --- | --- |
|  | Normal sample | Cancer Tissue | Pre-operative plasma (sample amount µL*) |  | Normal sample | Cancer Tissue | Pre-operative plasma (sample amount µL*) |
|  |  |  |  |  |  |  |  |
| GC1 | 27.52 | 23.60 | 27.66 (167) |  | - | 28.64 | - (833) |
| GC6 | 25.65 | 24.12 | 27.42 (167) |  | - | 26.70 | - (833) |
| GC10 | 28.22 | 24.62 | 29.72 (167) |  | - | 27.67 | - (833) |
| GC12 | 26.86 | 25.18 | 30.45 (167) |  | - | 26.85 | - (833) |
| GC18 | 24.12 | 23.71 | 27.13 (333) |  | - | 26.00 | - (333) |

* Sample amount of equivalent plasma employed for quantitative PCR

Ct, threshold cycles; -, not detected.
