# Supplementary material for: Monitoring circulating tumor DNA by analyzing personalized cancer-specific rearrangements to detect recurrence in gastric cancer
Source: Exp Mol Med. 2019 Aug 8;51(8):93. doi: 10.1038/s12276-019-0292-5 (PMC6802636; doi:10.1038/s12276-019-0292-5)
Supplement: Supplementary file 8 — Table S8 [file 12276_2019_292_MOESM8_ESM.doc]

Table S8. Quantitative measurement of ctDNA by digital droplet PCR

| Marker | Tissue | | ctDNA | | | | | |
| --- | --- | --- | --- | --- | --- | --- | --- | --- |
|  | Normal | Tumor | PreOP | PostOP | | | | |
|  |  |  |  | 1M | 3M | 6M | 9M | 12M |
| GC4 S4-6 | 0 | 301 | 28 | 16 | 17 | 13 | 3 | 26 |
| GC4 S4-7 | 0 | 354 | 19 | 15 | 10 | 5 | 9 | 11 |

The number of positive droplets is shown.

PreOP, pre-operative; PostOP, post-operative samples; 1M, 3M, 6M, 9M, and 12 M, plasma samples at 1, 3, 6, 9, and 12 months after surgery, respectively.
