# Supplementary material for: Monitoring circulating tumor DNA by analyzing personalized cancer-specific rearrangements to detect recurrence in gastric cancer
Source: Exp Mol Med. 2019 Aug 8;51(8):93. doi: 10.1038/s12276-019-0292-5 (PMC6802636; doi:10.1038/s12276-019-0292-5)
Supplement: Supplementary file 9 — Figure S1 [file 12276_2019_292_MOESM9_ESM.doc]

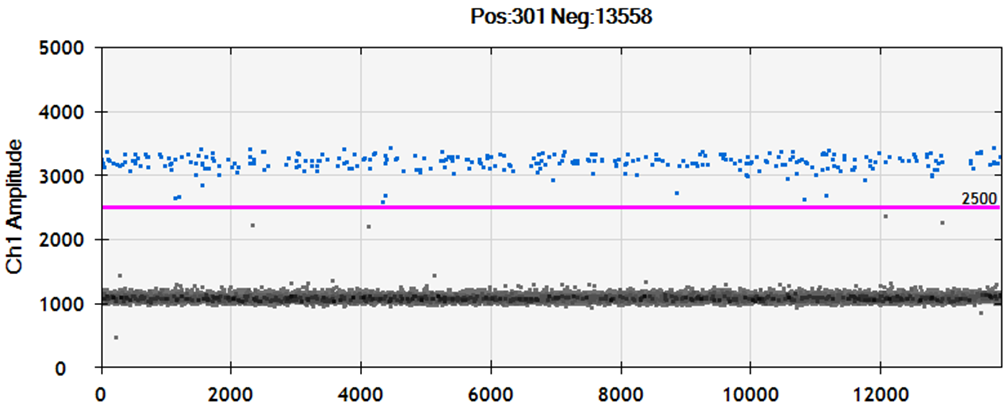

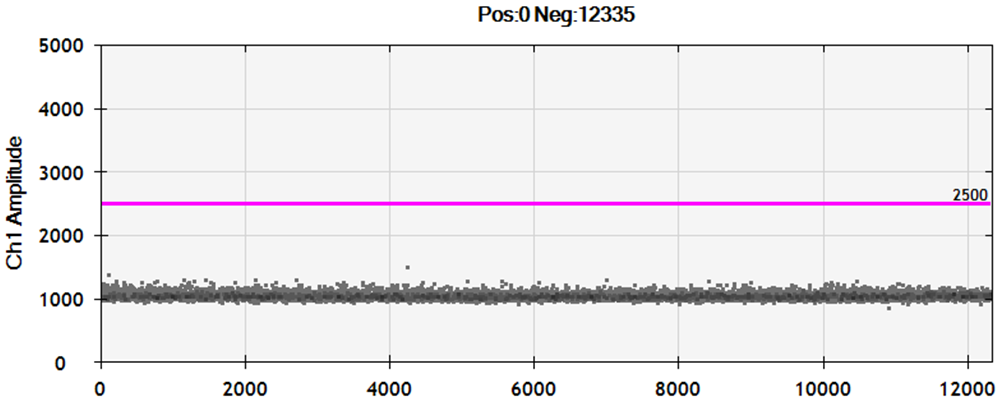

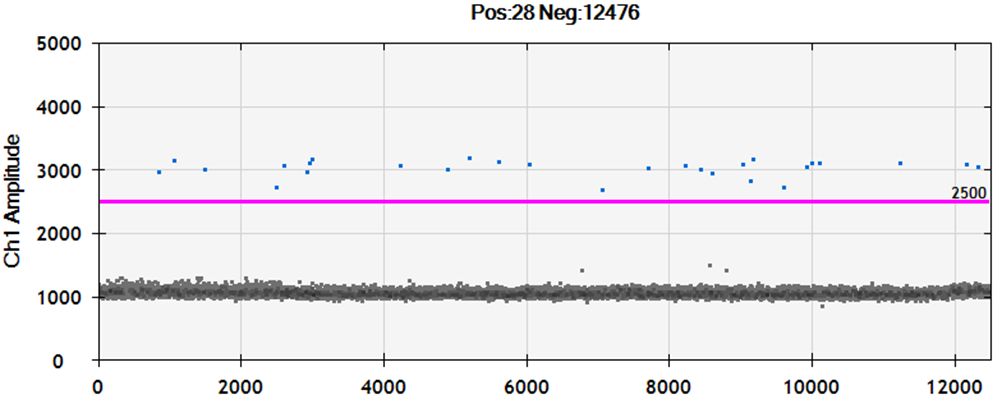

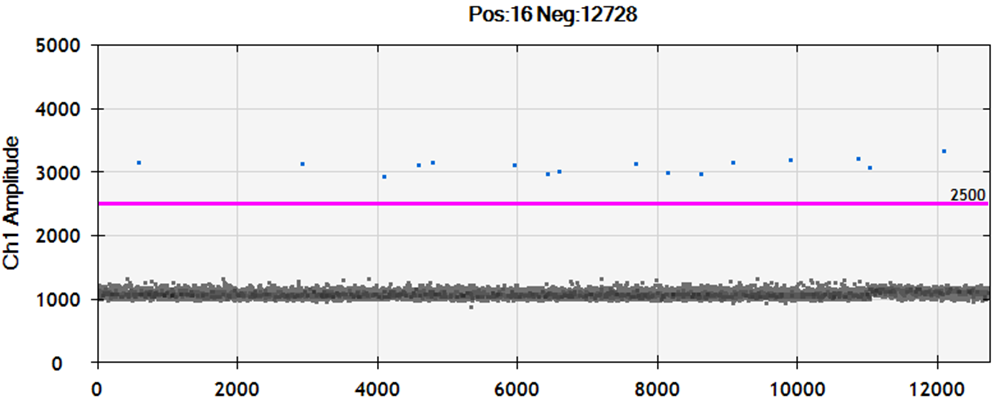

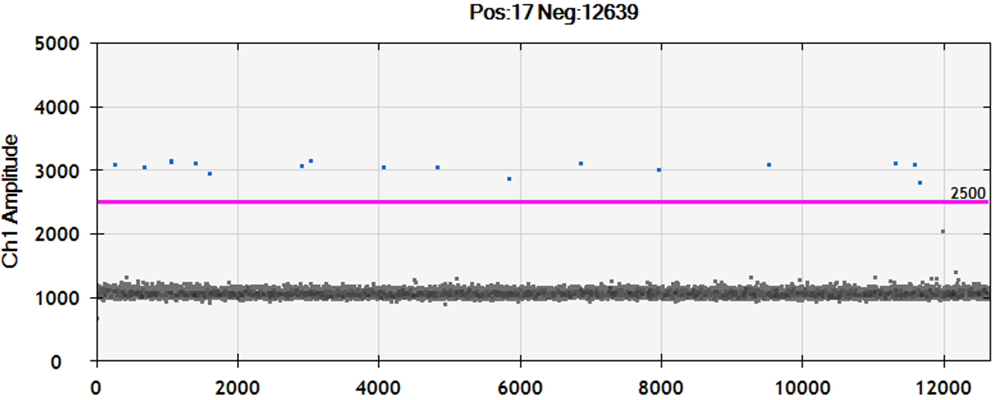

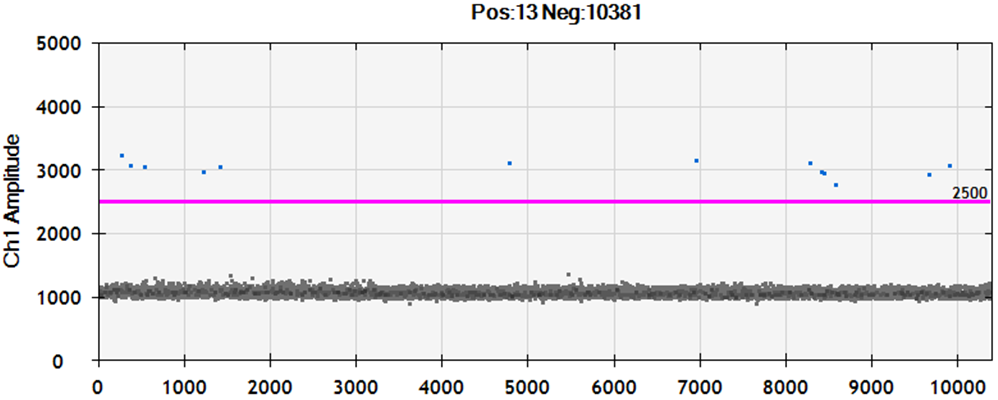

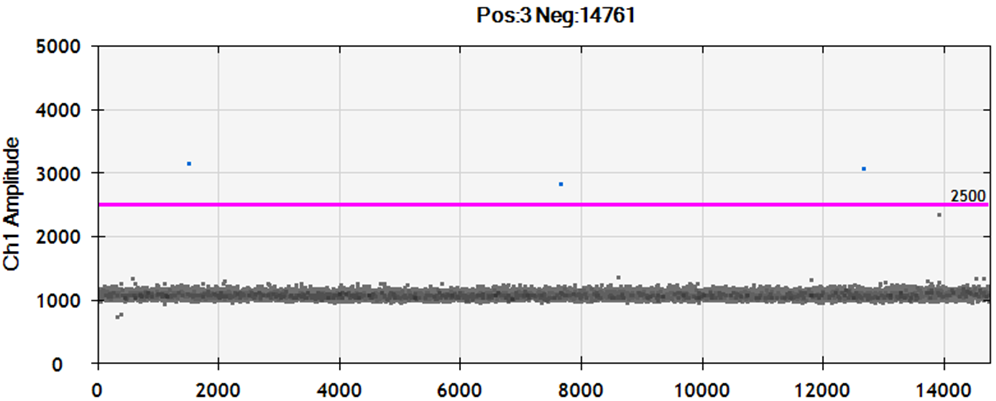

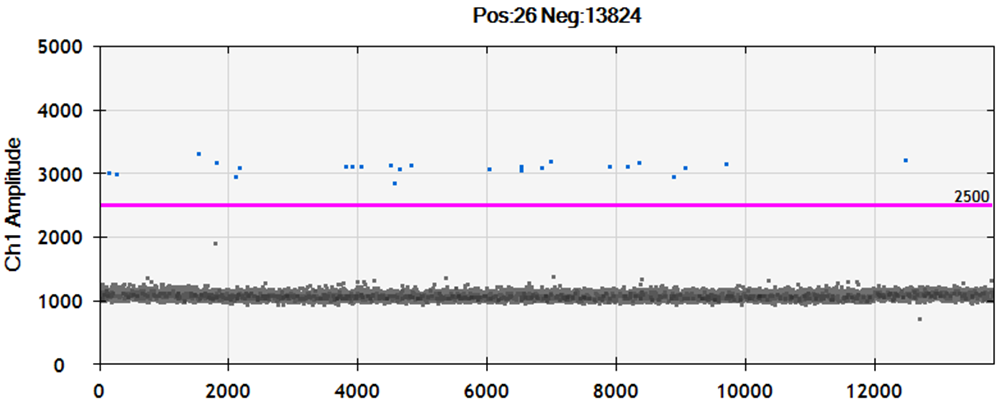

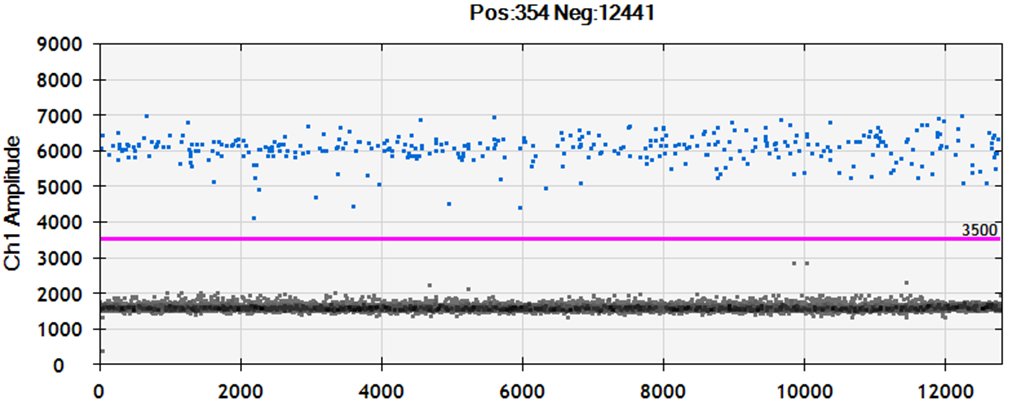

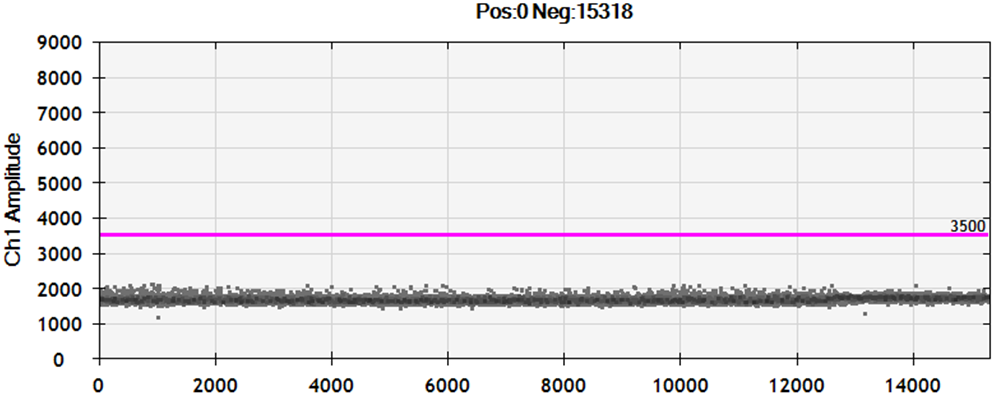

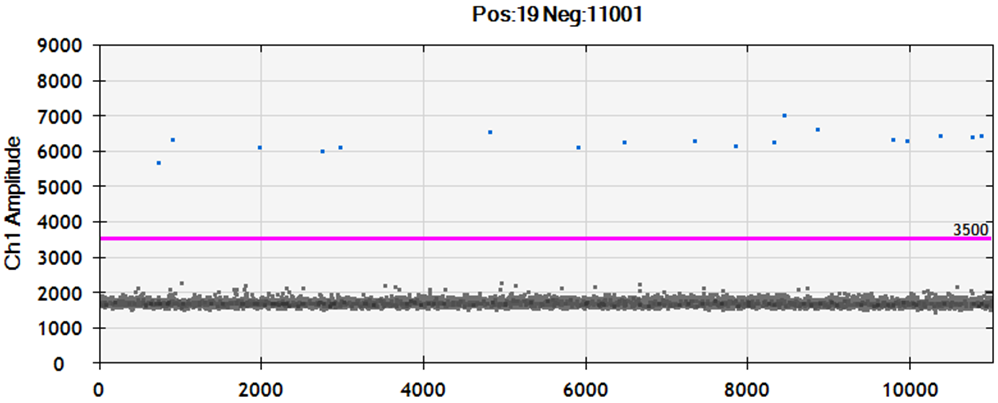

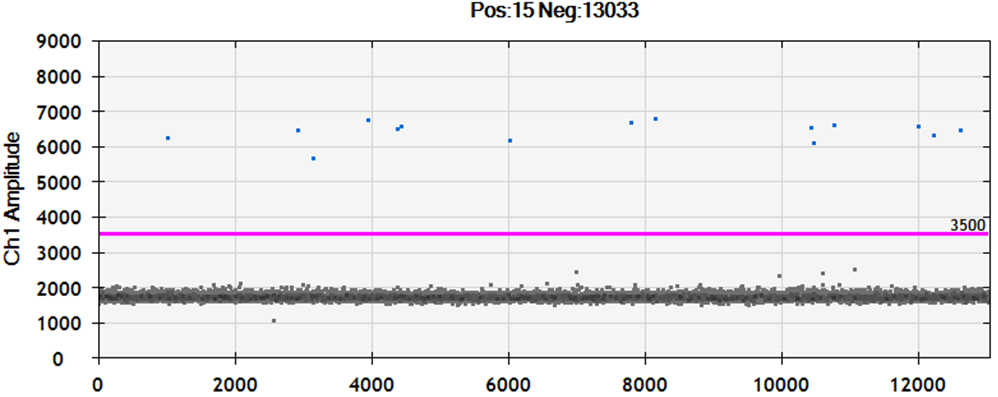

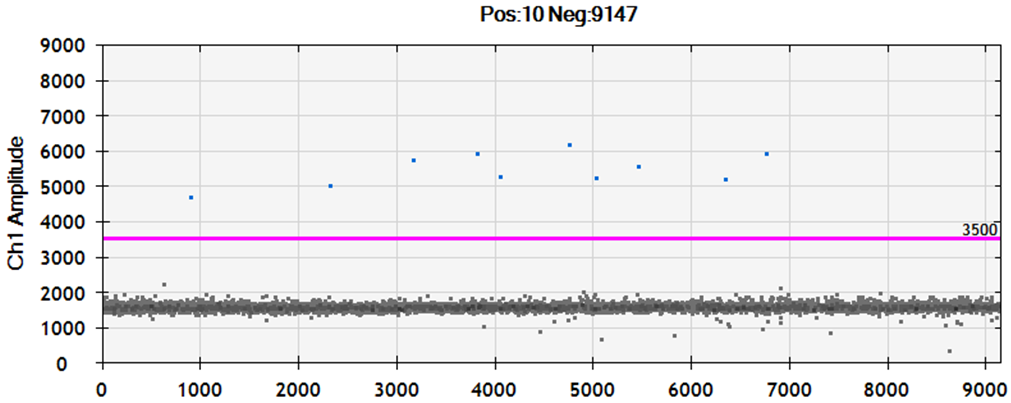

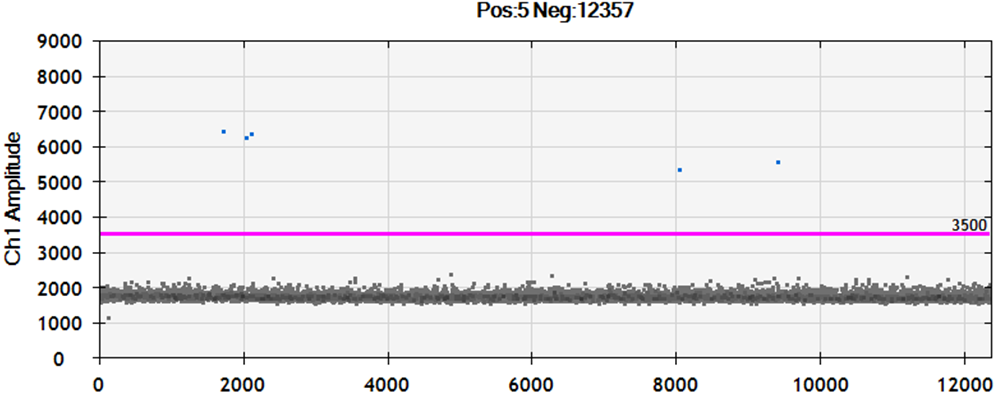

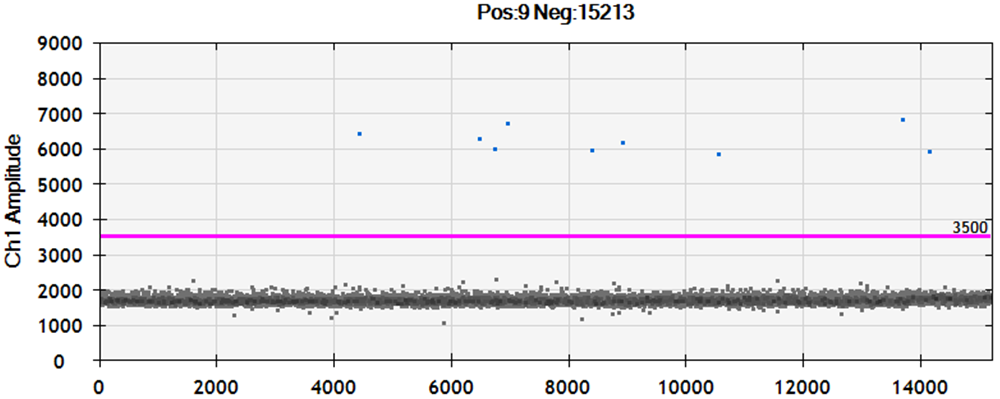

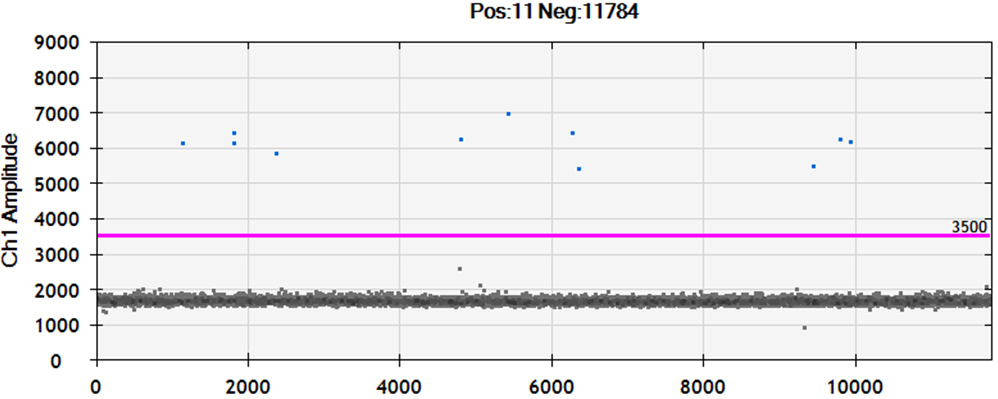


**Tumor**

**Normal**

**PreOP**

**1M**

**3M**

**6M**

**9M**

**12M**

GC4 S4-6

GC4 S4-7

Figure S1. Quantitative measurement of ctDNA in GC4 plasmas by digital droplet PCR (ddPCR). One-dimensional ddPCR plots for cancer-specific rearranged sequences are shown for the GC S4-6 and GC S4-7 markers. The blue dots are positive droplets, and the grey dots are negative ones. DNAs from Tumor (T) and Normal (N) tissues, and from pre-operative (PreOP) and post-operative (PostOP) plasma samples at 1, 3, 6, 9, and 12 months after surgery were employed. *GAPDH* data for control are not shown. ddPCR was performed by employment of 2 X ddPCR master mix (BioRad Laboratories, Hercules, CA, USA), and 10 μL of cell free DNA (equivalent to 333 μL of plasma sample) in a final reaction volume of 25 μL. Each reaction mixture was loaded into a sample well of an eight-channel disposable droplet generator cartridge (BioRad Laboratories). The emulsified samples were generated from a droplet generator (QX100; BioRad Laboratories) and then transferred onto a 96-well plate. After heat-sealing with a foil seal, the emulsified samples underwent a 2-step thermal cycling protocol in a C-1000 touch thermal cycler (BioRad Laboratories) as follows: 95℃ for 10 min, 40 cycles of 94℃ for 30 s and 55℃ for 60 s (ramp rate: 2℃ per second), and 98 ℃ for 10 min. The 96-well droplet PCR plates were loaded into a droplet reader (BioRad Laboratories) which automatically reads the droplets from each well of the plate. Analysis of the ddPCR data was performed with QuantaSoft analysis software version 1.7 (BioRad Laboratories). For detection of rearranged sites for the SC4 plasma samples in ddPCR, the following primers and probes were employed: SC4-6F (5′-TGG GCG GAG TAT AGG AGT TG), SC4-6R (5′-AGG AAT ATG TGT GTT GGG GG), and SC4-6Probe (5′-FAM-GCA GTG GGC AGG CTT ATC TA-BHQ1) for GC S4-6; GC4-7F (5′-ATG AGG CAC TCC AAG CAA AG), SC4-7R (5′-TGG GAG AGA AAG GAA GGT TTT), and GC4-7Probe (5′-FAM-CAG CAG CAA GAA TGC AAA AA-BHQ1) for GC S4-7. For detection of *GAPDH* as a reference gene in ddPCR, GAPDH-F (5′-TGC CTT CTT GCC TCT TGT CT), GAPDH-R (5′-AAT GAA GGG GTC ATT GAT GG), and GAPDH-Probe (5′-HEX-TCA CCA GGG CTG CTT TTA AC-BHQ1) were employed.
